# A Scalable and Robust Water Management Strategy for PEMFCs: Operando Electrothermal Mapping and Neutron Imaging Study

**DOI:** 10.1002/advs.202404350

**Published:** 2024-07-25

**Authors:** Linlin Xu, Panagiotis Trogadas, Shangwei Zhou, Shuxian Jiang, Yunsong Wu, Lara Rasha, Winfried Kockelmann, Jia Di Yang, Toby Neville, Rhodri Jervis, Dan J. L. Brett, Marc‐Olivier Coppens

**Affiliations:** ^1^ Centre for Nature‐Inspired Engineering Department of Chemical Engineering University College London London WC1E 7JE UK; ^2^ Department of Chemistry Aristotle University of Thessaloniki Thessaloniki 54124 Greece; ^3^ Electrochemical Innovation Lab Department of Chemical Engineering University College London London WC1E 7JE UK; ^4^ School of Electrical Engineering Southwest Jiaotong University Chengdu Sichuan 611756 China; ^5^ The Faraday Institution Quad One Harwell Science and Innovation Campus Didcot OX11 0RA UK; ^6^ Science and Technology Facilities Council Rutherford Appleton Laboratory ISIS Facility Harwell Oxford OX11 0QX UK

**Keywords:** electro‐thermal mapping, nature‐inspired, neutron radiography, polymer electrolyte membrane fuel cells, water management

## Abstract

Effective water management is crucial for the optimal operation of low‐temperature polymer electrolyte membrane fuel cells (PEMFCs). Excessive liquid water production can cause flooding in the gas diffusion electrodes and flow channels, limiting mass transfer and reducing PEMFC performance. To tackle this issue, a nature‐inspired chemical engineering (NICE) approach has been adopted that takes cues from the integument structure of desert‐dwelling lizards for passive water transport. By incorporating engraved, capillary microchannels into conventional flow fields, PEMFC performance improves significantly, including a 15% increase in maximum power density for a 25 cm^2^ cell and 13% for a 100 cm^2^ cell. Electro‐thermal maps of the lizard‐inspired flow field demonstrate a more uniform spatial distribution of current density and temperature than the conventional design. Neutron radiography provides evidence that capillary microchannels in the lizard‐inspired flow field facilitate the efficient transport and removal of generated liquid water, thereby preventing blockages in the reactant channels. These findings present a universally applicable and highly efficient water management strategy for PEMFCs, with the potential for widespread practical implementation for other electrochemical devices.

## Graphical abstract

This work presents a systematic nature‐inspired chemical engineering methodology to facilitate the design of flow fields for hydrogen fuel cells. Inspired by the passive water transport mechanism observed in desert lizards’ integument, a scalable water management strategy is devised. This approach significantly enhances water removal, uniformizes current density, improves resilience, and boosts fuel cell performance.

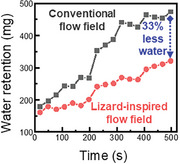

## 1 Introduction

Polymer electrolyte membrane fuel cells (PEMFCs) are environmentally friendly energy devices, garnering considerable attention due to their remarkable efficiency, low operating temperature, scalability, and potential for zero carbon emissions when renewable hydrogen is used.^[^ ^1^ ^]^ However, effective water management poses a significant challenge that must be overcome for the widespread adoption of PEMFCs.^[^ ^2^, ^3^ ^]^ The flow field is one of the critical components in PEMFCs, as it affects the electron and heat transfer, distribution of reactants, and removal of products.^[^ ^4^, ^5^ ^]^ If excess water, exceeding the required membrane hydration level and appearing from the electrochemical hydrogen oxidation or the humidification system, is not removed effectively, it accumulates in the catalyst layer, gas diffusion layer, or flow field.^[^ ^6^, ^7^ ^]^ This accumulation inhibits the transport of gas reactants to the catalyst layer, causing suboptimal performance of the PEMFC.^[^ ^8^ ^]^ However, insufficient water content induces membrane dehydration, thereby elevating ionic resistance and worsening voltage drop from Ohmic losses.^[^ ^3^ ^]^ Therefore, robust water management is essential for optimal performance, reliability, and longevity of PEMFCs.^[^ ^9^ ^]^

The general approach reported in the literature to overcome the problem of inefficient water removal in PEMFCs is based on empirical modifications in channel configuration (e.g., channel length, land width, and land/channel ratio).^[^ ^10^, ^11^, ^12^, ^13^ ^]^ This literature suggests flow fields with broader rib spacing, narrower channels, and shorter path lengths to improve the distribution of reactants. However, these modifications may be accompanied by decreased membrane hydration and conductivity, and higher pressure drops.^[^ ^10^, ^12^ ^]^

In our previous work, alternative routes inspired by physical phenomena and principles in nature that underpin properties also desired in fuel cells have been employed to overcome these limitations. For example, flow fields utilizing the fractal geometry of the lung were devised to address the challenge of reactant homogeneity across the catalyst layer.^[^ ^14^ ^]^ While improving mass transfer and minimizing pressure drop, such lung‐inspired, repeatedly branching flow fields were prone to flooding at very high (close to 100%) relative humidity (RH), and, thus, the fuel cell performance deteriorated. Subsequently, a novel water removal strategy was developed, involving the use of a laser‐drilled capillary array within the flow field region, which facilitated water supply or removal, depending on the local demand across the membrane electrode assembly (MEA).^[^ ^15^ ^]^ Nevertheless, the manufacturing and processing challenges associated with drilling micro holes through the entire thickness of the flow field plate to form capillary arrays remained formidable. As a result, a water management approach inspired by the structure and function of lizards was proposed. This method involved creating capillaries on the surface of a 1.2 mm thick graphite plate, which was then integrated within a lung‐inspired flow field with *N* = 4 branching generations. Despite the exceptional performance of this design in PEMFCs, it was only tested at a small scale of 10 cm^2^.^[^ ^16^ ^]^

**Scheme 1:**
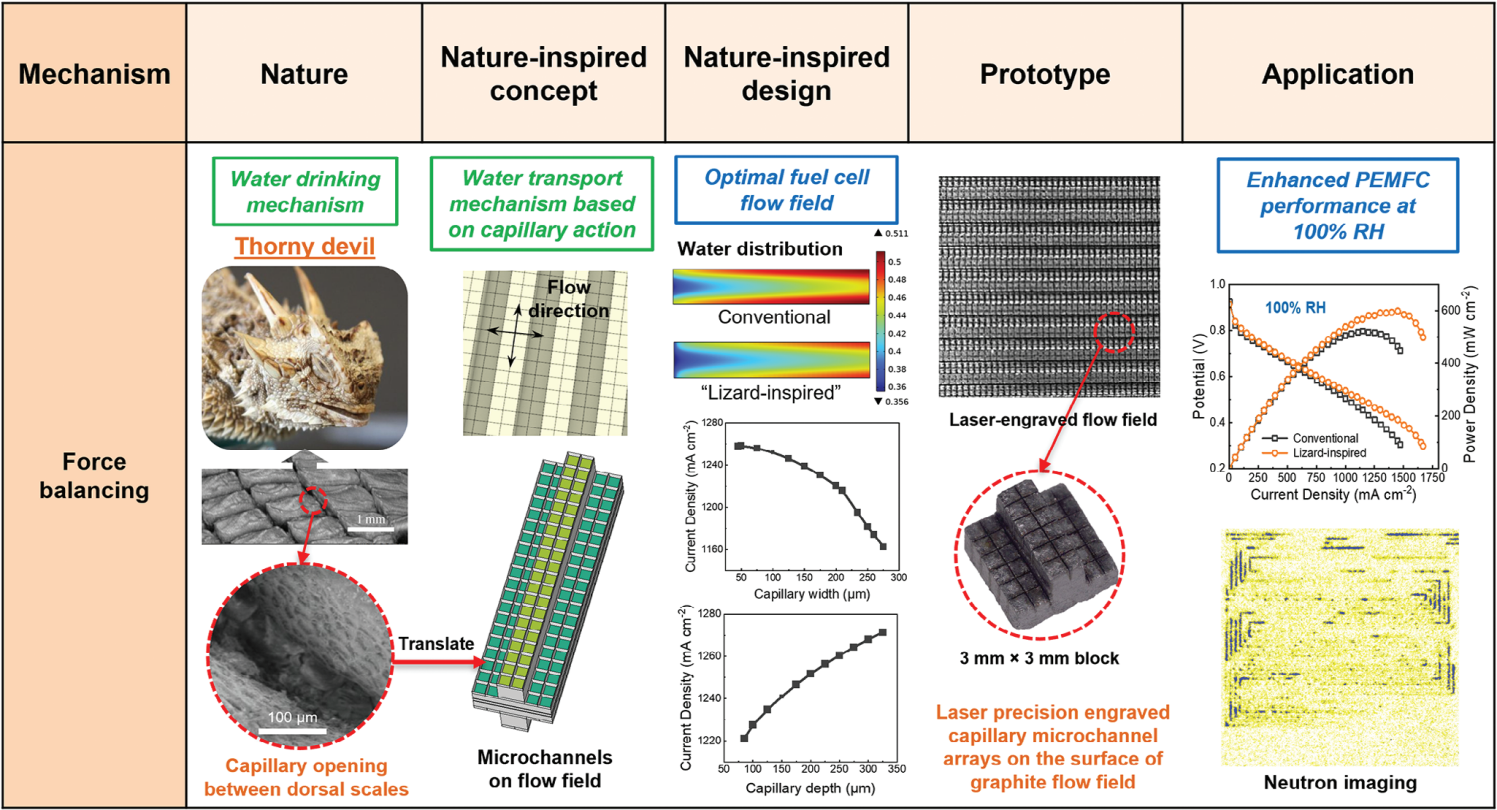
Implementation of the NICE approach in the design and engineering of a flow field for a PEMFC, taking inspiration from lizards residing in the desert. The images of the thorny devil and its integument are reproduced under the terms of the CC‐BY license.^[^ ^20^ ^]^ Copyright 2015, Royal Society.

In this work, an easier‐to‐implement technique for the lizard‐inspired water management strategy is proposed and its scalability up to 100 cm^2^ under the guidance of the nature‐inspired chemical engineering (NICE) methodology is investigated.^[^ ^17^ ^]^ The research process encompasses observation of nature, abstraction of nature‐inspired concepts, computationally assisted design, prototyping, and application (**Scheme** **1**).^[^ ^18^, ^19^ ^]^ Specifically, inspiration is drawn from several lizards residing in the desert, such as the Australian thorny devil and Texan horned lizard, which possess the ability to transport water passively (“Nature” in Scheme 1).^[^ ^16^ ^]^ These lizards feature an intricate network of interconnected capillary channels between overlapping scales of their integument. These channels, with diameters of ≈100–150 µm and depths of ≈50–200 µm, generate capillary pressure that facilitates passive water transport. The lizards collect water on their skin, which is then transported to the snout through a network of capillary channels. Once these capillary channels become saturated, water transport across them stops, and the lizards must ingest the water to maintain the flow of water to the snout.^[^ ^20^, ^21^ ^]^ The inherent characteristic of the water drinking mechanism employed by lizards, specifically the directed water transport through capillary action, persists unaltered even upon magnification. It is confirmed by quantitative analysis indicating the dominance of capillary forces over gravitational and viscous forces, governing the process of water transport within the scaled‐up structure (“Nature‐inspired concept”).^[^ ^16^ ^]^ This mechanism found in the lizard's unique skin structure is employed to create capillaries directly on the surface of conventional flow fields, which provides a more straightforward and efficient manufacturing process compared to the method of drilling micro holes through the entire thickness of the flow fields or assembling another thin graphite plate with capillary channels. A mathematical model is developed (Figures S1 and S2, Supporting Information), and simulations are conducted to estimate the impact of capillary channels (Figures S5–S7, Supporting Information) and calculate the optimal dimension of capillary channels (Figure S8, Supporting Information) in a fuel cell flow field (“Nature‐inspired design”), which is described in detail in the Section S1 (Supporting Information). Narrower and deeper channels have been found to notably improve PEMFC performance, as illustrated in Figure S8 (Supporting Information). However, when the width falls below 100 µm, further increases in current density are minimal, at <0.5%. Therefore, it is crucial to maximize capillary depth while keeping the capillary width within 100 µm. Such lizard‐inspired flow fields are created by a compact laser micromachining technique (“Prototype”). Specifically, this method involves creating capillaries directly on the surface of graphite flow fields, providing a more straightforward and efficient manufacturing process compared to drilling micro holes through the entire thickness of the flow fields or assembling additional thin graphite plate with capillary channels. The capillaries are engraved in both the ribs and channels of the graphite plates. The inclusion of channels alongside ribs serves critical functions in enhancing reactant distribution and water management within the cell. Channels are essential for ensuring even distribution of reactant gases, such as oxygen, across the MEA, thereby supporting consistent electrochemical reactions throughout the active area. Additionally, the engraved channels provide effective pathways for the drainage of liquid water from the cell, complementing the capillary action of ribs in preventing water accumulation and maintaining optimal cell performance. The water management ability of this design is investigated with PEMFC active area sizes of 25 and 100 cm^2^ to determine their scalability and effectiveness for practical applications. To this end, commercial flow fields are used as a baseline, and the performance is compared with the corresponding lizard‐inspired flow fields (“Application”). This study incorporates electrothermal mapping and water distribution analysis to offer a deeper understanding of current density distribution, thermal behavior, and water mapping in PEMFCs with different flow field designs.

## 2 Results and Discussion

### 2.1 PEMFC Performance and Impedance Analysis

#### 2.1.1 Polarization Curves and Dynamic Response Characteristics

**Figure 1:**
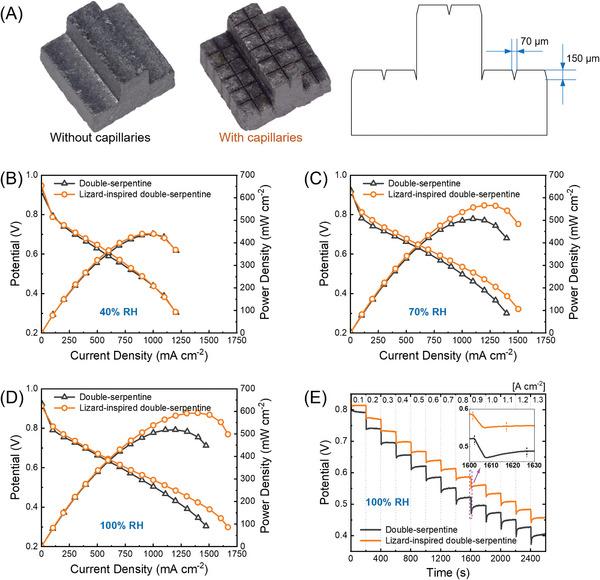
Comparison of morphology and PEMFC performance of double‐serpentine flow fields with and without capillaries. A) Side view and the dimensions of the capillary microchannels in a 3 × 3 mm^2^ block under the microscope. Polarisation and power density curves at B) 40% RH, C) 70% RH, and D) 100% RH, respectively. Each experiment was repeated at least three times to ensure that the results are statistically relevant. E) Dynamic response curves under changing load. PEMFC active area is 25 cm^2^.

Polarisation experiments were carried out to evaluate the performance of double‐serpentine flow fields with and without capillaries (**Figure** **1**). In what follows, the flow fields engraved with the capillary microchannels are called lizard‐inspired flow fields. At 40% RH (Figure 1B), condensation from the humidified stream across the active area is anticipated to be lower compared to higher RH levels, resulting in near flood‐free PEMFC operations for both flow fields. As the RH increases to 70% (Figure 1C) and 100% (Figure 1D), the lizard‐inspired flow field performs appreciably better than the conventional double‐serpentine flow fields, especially at high current density. At 100% RH, the lizard‐inspired double‐serpentine flow field achieves a peak power density of 598.3 mW cm^−2^ at 1450 mA cm^−2^. This represents a 15% improvement in peak power density over the conventional double‐serpentine flow field obtained at 1150 mA cm^−2^ (522.1 mW cm^−2^). Comparatively, the lizard‐inspired flow fields are less subject to mass transport issues, such as water flooding and reactant starvation, since the presence of horizontal and vertical micro flow paths (Figure 1A) results in more effective water transport, which restrains flooding during operation at higher RH. This improvement is supported by the stability test results (Figure S10, Supporting Information), which show minimal change in cell potential over 15 h. Furthermore, the capillary microchannels significantly enhance the transient stability of the double‐serpentine‐based fuel cell (Figure 1E). For example, after an increase from 800 to 900 mA cm^−2^, the transient cell voltage is lower than the steady‐state value, which is called undershoot^[^ ^22^, ^23^ ^]^ – the load change breaks the existing water balance. Specifically, the anodic ionomer rapidly dehydrates, as water migrates to the cathode side, carried by the protons. However, the lizard‐inspired flow field reaches a steady state more quickly than the conventional flow field, and this enhanced dynamic response performance comes from the ability of the nature‐inspired flow field to evenly redistribute water content, which is revealed by the electro‐thermal and water thickness mapping in the following sections.

#### 2.1.2 Electrochemical Impedance Spectroscopy

**Figure 2:**
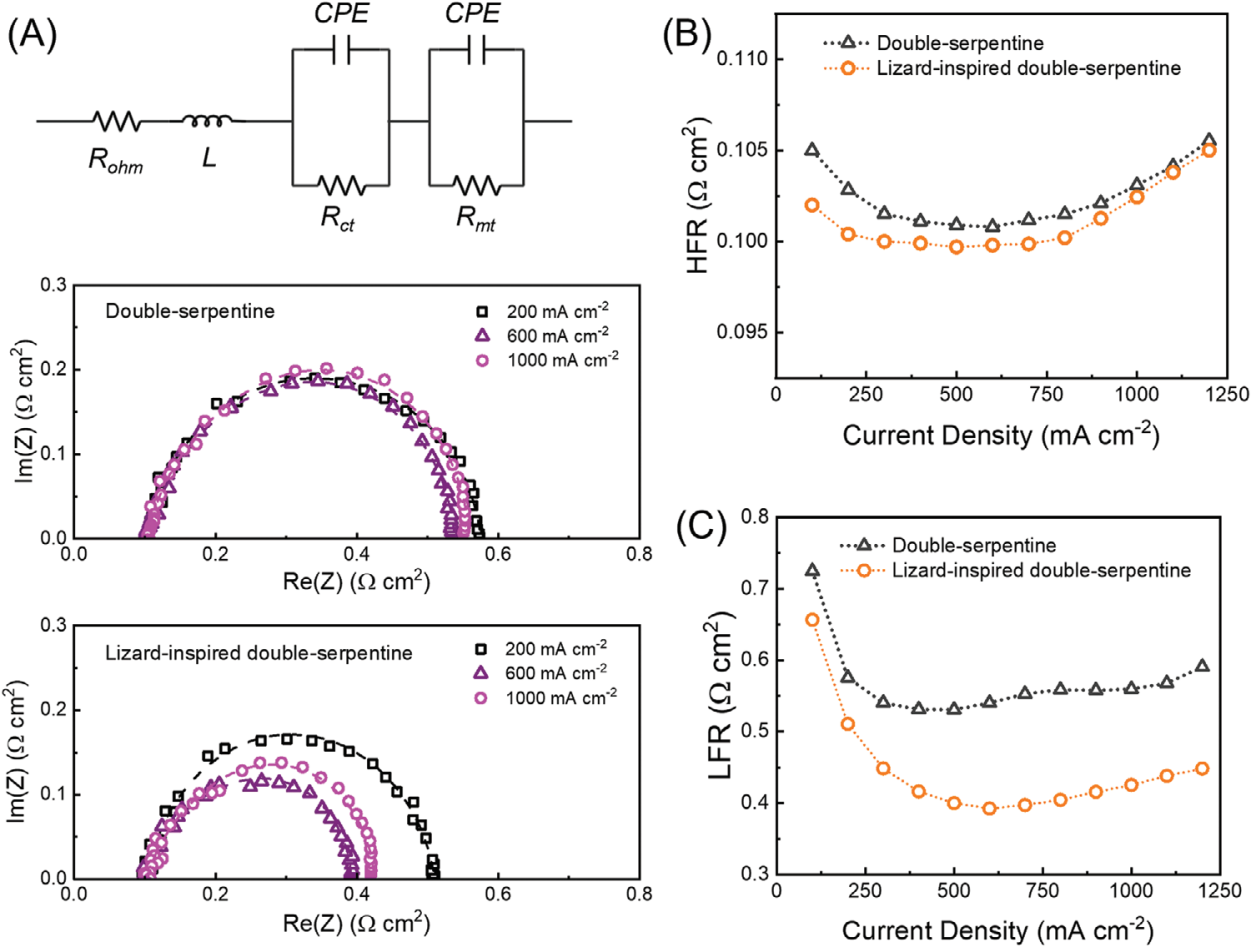
Comparison of the impedance of double‐serpentine flow fields with and without capillaries at 100% RH. A) The equivalent circuit model (on the top) for the EIS spectral analysis with two distinguishable arcs and Nyquist plots (double‐serpentine in the middle and lizard‐inspired at the bottom) taken at 200, 600, and 1000 mA cm^−2^, respectively. Notations: L – the pseudo inductance, R_ohm_ – Ohmic resistance (HFR), R_ct_ – charge transfer resistance, R_mt_ – mass transfer resistance, CPE – constant phase element.^[^ ^28^ ^]^ The dotted lines are fitted results. Changes in B) HFR and C) LFR with respect to current density. HFR and LFR are determined by the high‐frequency real axis intercept and low‐frequency real axis intercept in the Nyquist plot, respectively. PEMFC active area is 25 cm^2^.

Electrochemical impedance spectroscopy (EIS) provides information on the impedance of PEMFCs related to the electrochemical reaction rate and mass transport properties. The impedance spectra were fitted to an equivalent circuit model (**Figure** **2**A) to identify the contribution of each phenomenon to overall performance losses. At 200, 600, and 1000 mA cm^−2^, the total resistance of the lizard‐inspired double‐serpentine flow field (0.4–0.5 Ω cm^2^) is lower than that of the conventional double‐serpentine flow field (0.5–0.6 Ω cm^2^). The high‐frequency resistance (HFR) and low‐frequency resistance (LHR) at 100% RH are given in Figure 2B,C, respectively. HFR (or Ohmic resistance) is initially high at low current density and, as expected, gradually decreases as the membrane becomes more hydrated from liquid water generation.^[^ ^24^ ^]^ At high current densities (>800 mA cm^−2^), HFR begins to rise again because of the dehydration of the membrane, due to the high electro‐osmotic drag.^[^ ^25^ ^]^ Overall, the observed trends in HFR are due to well‐established phenomena common to both flow fields, indicating sufficient hydration in both cells.^[^ ^26^ ^]^ LFR corresponds to the total resistance developed in a cell. An initial decrease in LFR for both cells is spotted between 100 and 600 mA cm^−2^ as the cell transitions from the activation to the Ohmic region of operation. However, with an increase in current density to 800 mA cm^−2^ and further to 1200 mA cm^−2^, a gradual rise in LFR for the cells is observed, indicating increased resistances related to charge and mass transport due to cell flooding and reactant starvation.^[^ ^27^ ^]^ Although the commercial double‐serpentine geometry is known to achieve superior water removal, it still experiences local flooding in regions around the channel bends (Figure S13, Supporting Information), which may contribute to its higher overall system impedance.

### 2.2 Scalability

**Figure 3:**
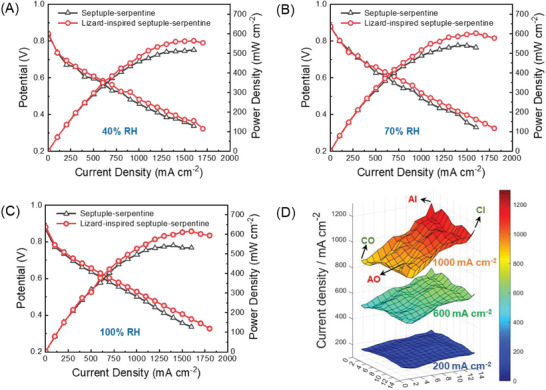
Comparison of polarisation and power density curves of septuple‐serpentine flow fields with and without capillaries at A) 40% RH, B) 70% RH, and C) 100% RH, respectively. Each experiment was repeated at least three times to ensure that the results are statistically relevant. D) Current density distribution mapping of lizard‐inspired septuple‐serpentine flow field at 100% RH under 200, 600, and 1000 mA cm^−2^. Current density distribution was obtained via S++ simulation for each sensor plate and subsequently plotted in MATLAB. AI, AO, CI, and CO indicate the anode inlet, anode outlet, cathode inlet, and cathode outlet, respectively. PEMFC active area is 100 cm^2^.

To maintain PEMFC performance without excessive pressure drop in the 100 cm^2^ double‐serpentine flow field, more channels of serpentine design, leading to a septuple‐serpentine flow field, were used for the 100 cm^2^ fuel cell.^[^ ^29^ ^]^ **Figure** **3**A–C show the polarisation curves for septuple‐serpentine flow fields, with and without capillaries, when the size of the PEMFC is 100 cm^2^. The results reveal that the lizard‐inspired septuple‐serpentine flow field outperforms the traditional one, with a 9%, 11%, and 13% improvement in peak power density at 40% RH, 70% RH, and 100% RH, respectively. This enhancement can be attributed to applying lizard‐inspired capillary microchannels that improve the PEMFCs' performance, irrespective of the flow field design and fuel cell scale.

The higher current densities observed in the 100 cm^2^ cell configuration than in the 25 cm^2^ cell configuration are due to the larger number of serpentine channels, increasing the surface area available for electrochemical reactions and the mass transport of reactants and products. However, it also results in higher water production during PEMFC operation. The larger active area and longer flow paths increase the residence time of reactants within the flow channels, leading to a more completed reaction and higher water production.^[^ ^30^, ^31^ ^]^ This also explains why lizard‐inspired flow fields perform better than traditional flow fields at a larger surface area of 100 cm^2^ at 40% RH but not when the cell size is 25 cm^2^.

### 2.3 Coupled Analysis of Current, Temperature, and Water Distribution

Figure 3D illustrates a notable disparity in the current distribution at average current densities of 200, 600, and 1000 mA cm^−2^, with the distribution becoming less uniform at higher current densities. To gain more insights into these discrepancies, evaluating the electro‐thermal (current density and temperature) mapping and the water distribution in different regions within a polarisation curve, including activation, Ohmic, and mass transport regions, is essential.^[^ ^22^, ^32^, ^33^ ^]^

**Figure 4:**
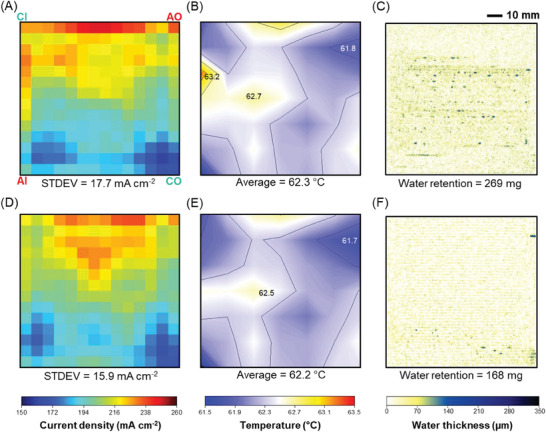
Comparison of A,D) current, B,E) temperature, and C,F) water distribution at 200 mA cm^−2^ of septuple‐serpentine flow fields (D–F) with and (A–C) without capillaries. Current density and temperature distribution were obtained via S++ simulation for each sensor plate and subsequently plotted in MATLAB. For water distribution analysis, a set of ten images was filtered to remove outliers and then averaged using the median. AI, AO, CI, and CO indicate the anode inlet, anode outlet, cathode inlet, and cathode outlet, respectively. PEMFC active area is 100 cm^2^.

At an average current density of 200 mA cm^−2^, both septuple‐serpentine flow fields with and without capillaries exhibit a relatively uniform distribution of current density (**Figure** **4**A,D), temperature (Figure 4B,E) and water (Figure 4C,F) across the entire active area. This uniformity is attributed to the ease of diffusion of reactants through the thin diffusion layer, facilitating even distribution of the electrochemical reactions.^[^ ^34^ ^]^ The overall reaction rate is limited by the availability of reactants, and the low current density is not enough to generate significant amounts of heat, resulting in a low temperature gradient across the active area. The water distribution is usually stable, with water being consumed at a relatively constant rate to keep the polymer electrolyte membrane hydrated. The lizard‐inspired septuple‐serpentine flow field generally shows greater uniformity than the conventional septuple‐serpentine flow field. This is demonstrated by the lower standard deviation (STDEV) of the current density distribution, which is 15.9 mA cm^−2^ (Figure 4D) for the lizard‐inspired septuple‐serpentine flow field compared to 17.7 mA cm^−2^ (Figure 4A) for the septuple‐serpentine flow field without capillaries, despite both maintaining the same average current density. The improvement is attributed to the enhanced water management ability. The neutron radiography image (Figure 4F) further confirms this by revealing a thin layer of water covering the active area of the lizard‐inspired flow field, ensuring the hydration of the membrane. In contrast, the unmodified flow field exhibits more visible water droplets (Figure 4C), indicating water accumulation and ineffective water removal from the electrode surface.

**Figure 5:**
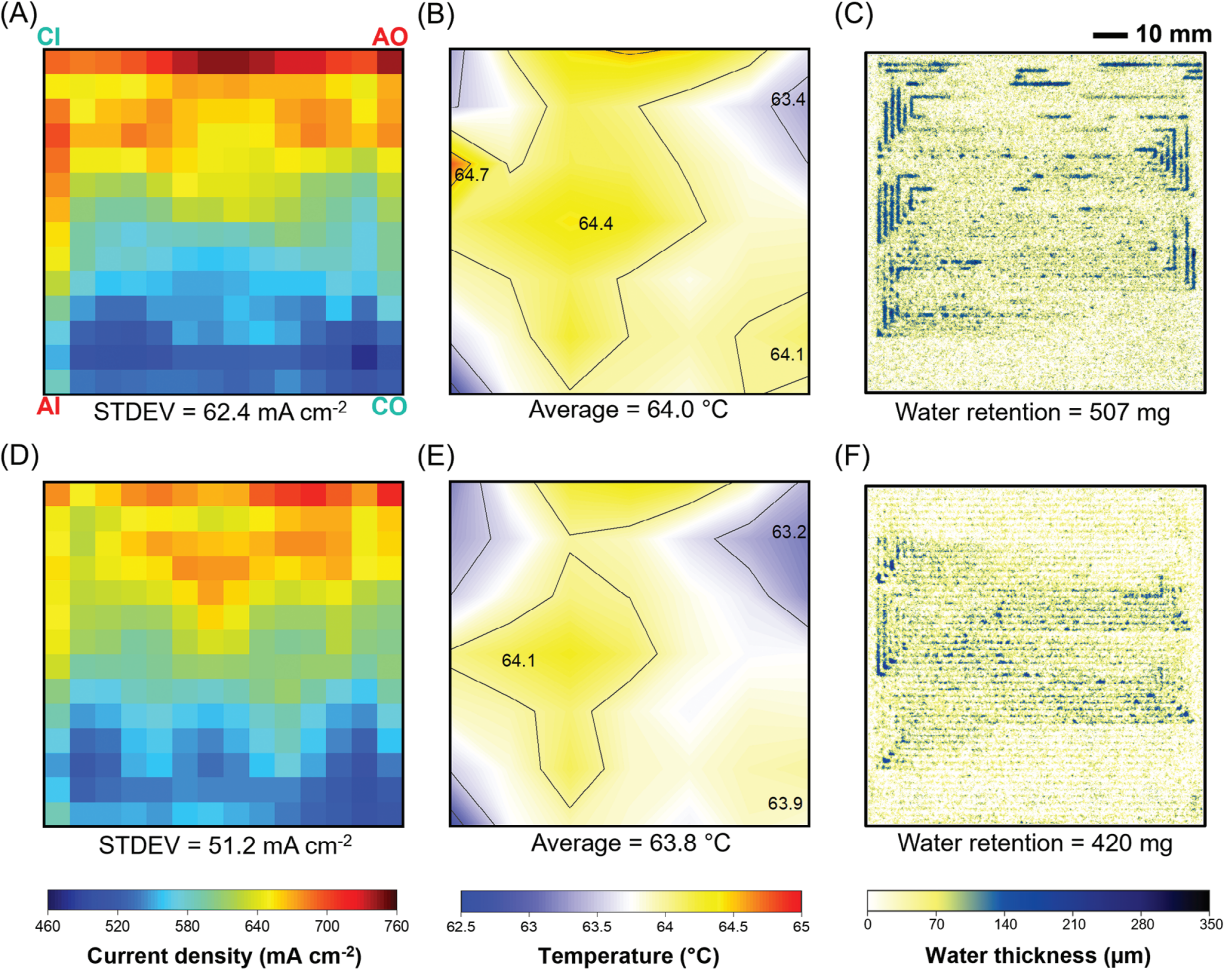
Comparison of A,D) current, B,E) temperature, and C,F) water distribution at 600 mA cm^−2^ of septuple‐serpentine flow fields (D–F) with and (A–C) without capillaries. Current density and temperature distribution were obtained via S++ simulation for each sensor plate and subsequently plotted in MATLAB. For water distribution analysis, a set of ten images was filtered to remove outliers and then averaged using the median. AI, AO, CI, and CO indicate the anode inlet, anode outlet, cathode inlet, and cathode outlet, respectively. PEMFC active area is 100 cm^2^.

As the average current density increases to 600 mA cm^−2^ (**Figure** **5**), the electro‐thermal mapping (Figure 5A,B,D,E) and water distribution (Figure 5C,F) become less uniform. The cathode inlet has a relatively higher current density than the cathode outlet (Figure 5A,D). The lizard‐inspired flow field provides a uniform distribution of reactants over the active area of the cell, avoiding huge, localized variations in the reaction rate.^[^ ^35^ ^]^ As the electrochemical reaction rate increases, so does the production of water.^[^ ^36^ ^]^ The water retention of the septuple‐serpentine flow field without capillaries at 600 mA cm^−2^ is 507 mg (Figure 5C), calculated according to the Beer–Lambert law, and up from 269 mg at 200 mA cm^−2^.^[^ ^37^ ^]^ Liquid water begins to accumulate around channel bends due to the decreasing channel‐to‐channel pressure gradient near the bends and flow instability, which indicates that the generated water is not removed in time.^[^ ^33^, ^38^ ^]^ In contrast, the water content under the lizard‐inspired septuple‐serpentine flow field is much lower (420 mg) (Figure 5F).

**Figure 6:**
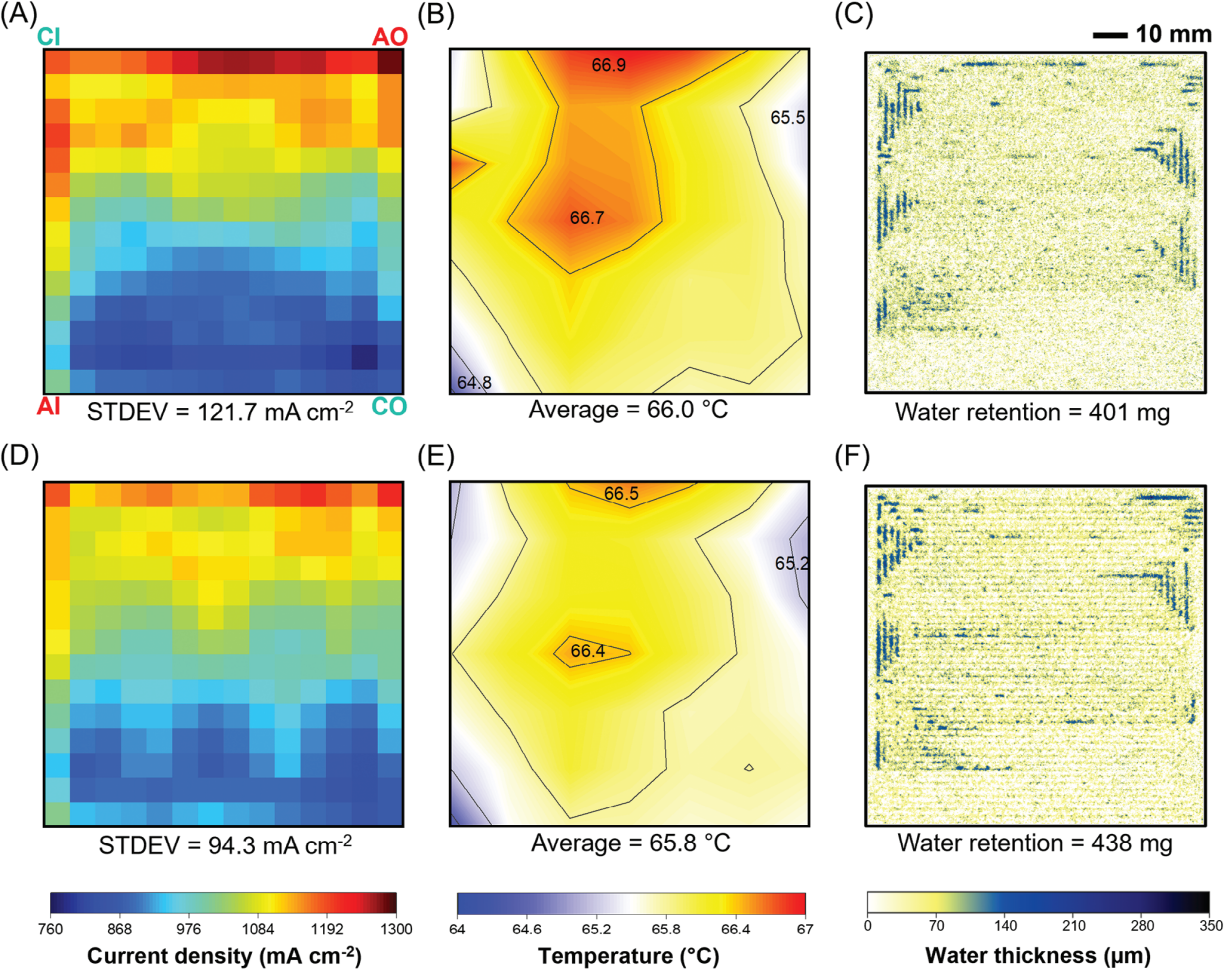
Comparison of A,D) current, B,E) temperature, and C,F) water distribution at 1000 mA cm^−2^ of septuple‐serpentine flow fields (D–F) with and (A–C) without capillaries. Current density and temperature distribution were obtained via S++ simulation for each sensor plate and subsequently plotted in MATLAB. For water distribution analysis, a set of ten images was filtered to remove outliers and then averaged using the median. AI, AO, CI, and CO indicate the anode inlet, anode outlet, cathode inlet, and cathode outlet, respectively. PEMFC active area is 100 cm^2^.

At an average current density of 1000 mA cm^−2^ the current density distribution becomes more uneven, as depicted in **Figure** **6**A,D. The heat produced during the reactions increases the temperature of the fuel cell, creating a significant temperature gradient (Figure 6B,E). The center of the electrode becomes much hotter than the edges, and the temperature at the cell inlet is lower than that at the outlet due to the high concentration of electrochemical reactions generating a large amount of heat.^[^ ^36^ ^]^ When compared to the neutron images at 600 mA cm^−2^, it is observed that the septuple‐serpentine flow field without capillaries generates less water (401 mg) at 1000 mA cm^−2^ (Figure 6C). This is because the high cell temperature can lead to the evaporation of water, and a high gas velocity across the channel is beneficial for carrying water out.^[^ ^39^, ^40^ ^]^ However, the lizard‐inspired flow field can generate a comparable quantity of water (Figure 6F), indicating improved tolerance to dehydration at high current density.^[^ ^39^, ^40^ ^]^ It is also notable that the water retention for the lizard‐inspired flow field is similar to that without capillaries for the septuple flow fields at 1000 mA cm^−2^. This similarity arises from the relatively low pressure drop of both designs (Figure S14, Supporting Information), with the septuple‐serpentine flow field without capillaries having a pressure drop of 15 kPa and the lizard‐inspired septuple‐serpentine flow field having a pressure drop of 12 kPa, which are insufficient for efficient water removal.^[^ ^41^, ^42^ ^]^ The lower maximum temperatures of the lizard‐inspired flow field also indicate its potential to dissipate heat generated during the electrochemical reaction to prevent localized hotspots, which lead to material degradation and reduced cell performance.^[^ ^11^ ^]^ If there were no cooling flow channel placed on the back of the anode flow field, the temperature distribution difference would be much more pronounced.

When the reactants are humid, it can be challenging to distinguish between the water produced by the electrochemical reaction and the water present in the reactants themselves. To accurately measure the water generated during PEMFC operation and understand the water management behavior of the 100 cm^2^ flow fields, a galvanostatic measurement at 400 mA cm^−2^ was performed at 40 °C without gas humidification.

**Figure 7:**
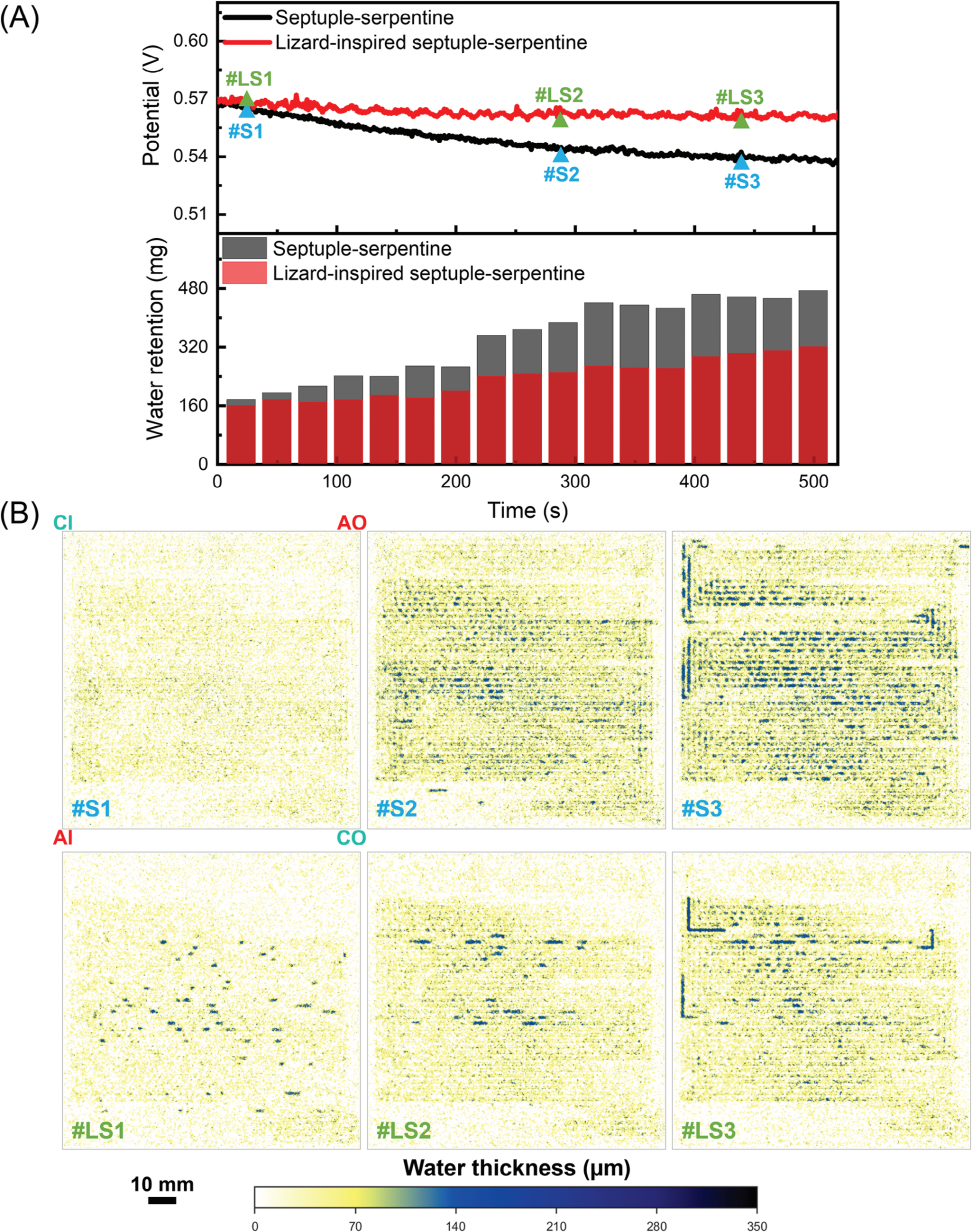
A) Transient changes in potential and water retention during galvanostatic operation of septuple‐serpentine flow field with and without capillaries at 400 mA cm^−2^, as well as at B) corresponding neutron images of the water thickness distribution. S and LS in this diagram indicate the septuple‐serpentine without capillaries and lizard‐inspired septuple‐serpentine flow fields, respectively. Experiments were conducted at a cell temperature of 40 °C using dry hydrogen and dry air at a stoichiometric ratio of 2 and 3, respectively. AI, AO, CI, and CO indicate the anode inlet, anode outlet, cathode inlet, and cathode outlet, respectively. PEMFC active area is 100 cm^2^.

During the current hold (**Figure** **7**A), the voltage of the lizard‐inspired flow field remains almost constant, while the septuple‐serpentine flow field without capillaries experiences a 5% voltage drop after 500 s. The generation of water was observed in real time and shown in Figure 7B. At the beginning of the current hold, most of the liquid water appears as droplets and films (#S1, #LS1), and both flow fields have similar voltage and water generation levels. The lizard‐inspired flow field has more distinct droplets near the center of the active area, indicating a more intense electrochemical reaction. After maintaining the current for 290 s, more water accumulates in the cell, resulting in a larger difference in water generation between the two flow field designs (386 mg in #S2 and 248 mg in #LS2). The counter‐current flow orientation, where dry air and hydrogen move in opposite directions, induces a drier environment in the upper and lower regions of the active area due to evaporation and back‐diffusion mechanisms, respectively.^[^ ^43^ ^]^ When reaching #S3 (457 mg), the conventional septuple‐serpentine flow field exhibits significant water columns at both the bends and center, indicating poor ability to remove water. In contrast, the lizard‐inspired flow field produces considerably less water at #LS3 (302 mg), and the water column at the bends can be carried forward horizontally, thereby taken out of the PEMFC. The flow field design inspired by the lizard exhibits superior water management performance without incurring increased pressure drop. This is illustrated in Figure S14 (Supporting Information), where the pressure drops for the lizard‐inspired flow field are lower compared to the unmodified flow field under different conditions.

## 3 Conclusion

In this study, a water management strategy inspired by the passive water transport mechanism of desert‐dwelling lizards, was employed for 25 cm^2^ double‐serpentine and 100 cm^2^ septuple‐serpentine flow fields in PEMFCs. The beneficial traits of the lizard integument were translated to capillary microchannels among the flow fields to improve the liquid water removal efficiency in PEMFCs.

The results showed that the integration of capillary microchannels in the lizard‐inspired flow fields resulted in over 10% improvement in maximum power density compared to unmodified flow fields at 100% RH for both 25 and 100 cm^2^ fuel cells, confirming the feasibility and scalability of this design. The capillary microchannels also played a significant role in enhancing transient stability by removing liquid water before it could form droplets in the channels. Electro‐thermal mapping revealed that the lizard‐inspired flow field results in more homogeneous current and temperature distributions. At a current density of 1000 mA cm^−2^, the standard deviation of current density distribution was reduced from 121.7 mA cm^−2^ in the septuple‐serpentine flow field without capillaries to 94.3 mA cm^−2^ in the lizard‐inspired septuple‐serpentine flow field. Neutron radiography images validated the effectiveness of the lizard‐inspired flow fields in maintaining membrane hydration and reducing water accumulation. At 400 mA cm^−2^ under dry reactant gas conditions, a lizard‐inspired septuple‐serpentine flow field generated 155 mg less liquid water compared to traditional septuple‐serpentine flow field after 500 s.

Overall, the lizard‐inspired flow field demonstrated promising results in enhancing the performance, stability, water management, and mass transport properties of PEMFCs, making the fuel cell work robustly under a wide range of RH that poses less challenges for the humidification control strategy. These findings hold significant potential for the development of more efficient and intensified fuel cell technologies. However, to ensure the commercial viability of these designs, future work should involve comprehensive durability testing by subjecting the PEMFCs to a variety of operational conditions and stress factors, such as temperature fluctuations, humidity variations, and prolonged operational cycles, to assess their long‐term performance and reliability.

## 4 Experimental Section

### Flow Field Fabrication

A double‐serpentine flow field design (Figure S9A, Supporting Information) was used for the 25 cm^2^ cell, while a septuple‐serpentine (Figure S9B, Supporting Information) was used for the 100 cm^2^ cell to avoid a high‐pressure drop.^[^ ^29^ ^]^ The flow fields were machined from 6 mm thick graphite plates (Schunk) using a CNC machine (Roland 40A), with channel width, spacing, and depth of 1 mm. A Compact Laser Micromachining System (Oxford Lasers) was used to engrave the interconnected capillary arrays on the surface of cathode flow fields. The dimensions of the capillary channels were based on computer simulations in Figure S8 (Supporting Information) to maximize the capillary depth while ensuring that the capillary width was kept within 100 µm. The 101 × 101 and 201 × 201 capillary array networks were engraved into 25 and 100 cm^2^ flow fields, respectively. A capillary spacing of 500 µm was selected for both flow fields based on the equivalence to half the width of the flow channel and rib. This choice enables the evaluation of the conceptual impact, while mitigating potential complexities and costs associated with the utilisation of additional capillary arrays. Digital microscope photographs (Keyence fully integrated head VHX‐7100) revealed that the microchannels were spread uniformly across the graphite plate and had dimensions of ≈70 µm width and ≈150 µm depth (Figure 1A). After laser engraving, the graphite surfaces were washed with a small amount of deionized water and dried with an air gun to remove debris. To clarify, laser engraving was specifically conducted on the cathode flow field, while the anode flow field remained without engraving.

### Membrane Electrode Assembly (MEA) Preparation

The MEAs were prepared in‐house by hot pressing a Nafion 212 (DuPont) membrane sandwiched between gas diffusion electrodes (HyPlat) using a thermal press (Carver Auto Series Plus) at 140 °C for 3 min under an applied pressure of 400 psig. The catalyst layers had a platinum loading of 0.4 mg_Pt_ cm^−2^ at both the cathode and anode.

### Single Cell Test

A commercial Scribner 850e fuel cell test station (Scribner Associates) was connected to the 25 cm^2^ cell. The stoichiometry of hydrogen (anode inlet gas) and air (cathode inlet gas) were kept constant at 2 and 3, respectively. Inlet reactants were humidified using bubbler type humidifier tanks. The inlet gas RH of the anode and cathode was varied between 40%, 70%, and 100%, and the cell temperature was set to 60 °C. The outlet back pressure of the anode and cathode remained at atmospheric pressure. Transient changes in cell potential were recorded over a period of 200 s at each current density for each current hold experiment. The Greenlight G60 (Greenlight Innovation) test bench was employed to test the 100 cm^2^ cell with the same operating conditions as the 25 cm^2^ cell configuration. The cooling system was custom designed, using deionized water as the coolant, which was kept at a temperature of 60 °C and circulated through the back of the anode flow field. An S++ (S++ Simulation Services) electro‐thermal mapping plate^[^ ^34^ ^]^ was sandwiched between the 100 cm^2^ cathode flow field and cathode current collector, consisting of an array of 14 × 14 integrated shunt resistors to study the dynamic changes in current density distribution and a 7 × 7 array of temperature sensors for surface temperature distribution measurement. Current and temperature measurements were made simultaneously, and their respective sensors were located on the same sensor plate, made of a printed circuit board with gold‐coated contact segments. All experiments in this study were conducted using a fresh MEA and the same initial conditioning process to ensure identical starting conditions. Each experiment was repeated at least three times to ensure that the results were statistically relevant.

### Electrochemical Impedance Spectroscopy (EIS) Measurements

EIS was performed using a Gamry Reference 3000 and Gamry Reference 30 K Booster (Gamry Instruments). Prior to the impedance measurement, the fuel cell was conditioned at each constant current to reach the steady state. Data points were recorded at a frequency range of 10 kHz to 0.1 Hz (10 points/decade), and the alternating current (AC) modulation amplitude was kept at 10% of the direct current (DC) input signal to ensure a linear system response.

### Neutron Imaging

Neutron radiography was conducted at the neutron imaging facility Imaging and Materials Science (IMAT) at the Rutherford Appleton Laboratory, UK.^[^ ^44^ ^]^ A through‐plane orientation was employed to visualize the distribution of water throughout the entire active area of the PEMFCs. A neutron camera box equipped with a ZnS/LiF scintillation screen and an ANDOR sCMOS Zyla 4.2 module was used for neutron imaging. In the case of a 25 cm^2^ cell configuration, an imaging field of view measuring 112.6 × 112.6 mm^2^ was achieved, with a pixel size of 55 µm and effective spatial resolution of the setup of 0.15 mm. For a 100 cm^2^ cell configuration, a larger imaging field‐of‐view of 210.9 × 210.9 mm^2^ with a pixel size of 103 µm and a spatial resolution of 0.23 mm was attained. The exposure time was set to 30 s for each image to ensure high‐quality data on the premise of capturing the dynamic changes of water. Images were taken during cell operation and normalized to a dry image taken at the start of each experiment to isolate liquid water, and the water thickness was determined using the Beer–Lambert law.^[^ ^37^, ^43^ ^]^ The attenuation coefficient of neutrons in liquid water was measured for the given setup to be 5.3 cm^−1^. Details are provided in the Section S4 (Supporting Information). The mass of accumulated liquid water in the PEMFCs was determined by integrating the localized water thickness across the entire active area from the averaged neutron image to evaluate the impact of flow fields on total liquid water content.

### Statistical Analysis

The data for current density, potential, and power density were acquired using the Scribner 850e fuel cell test station and the Greenlight G60 test bench. Resistance measurements were directly obtained from Gamry Instruments. Data on current density distribution and temperature distribution were provided by S++ Simulation Services for each sensor plate and subsequently plotted in MATLAB R2022b. Neutron images were captured at IMAT and processed using ImageJ. For water distribution analysis, as depicted in Figures 4, 5, 6, each set of ten images was filtered to remove outliers and then averaged using the median. The averaged dark‐field image was subtracted from the resultant image, which was then normalized against an averaged dry image to obtain the final image. Greyscale values were converted into water thickness using the Beer–Lambert law, and the results were plotted in MATLAB R2022b. The mass of accumulated liquid water was determined by integrating the localized water thickness across the entire active area using MATLAB R2022b. Pressure drop data were derived by subtracting the pressure readings at the inlet and outlet. These pressure readings were obtained using a Data Acquisition (DAQ) system with programmable software LabVIEW.

## Conflict of Interest

The authors declare no conflict of interest.

## Supporting information

Supporting Information

## Data Availability

The data that support the findings of this study are available from the corresponding author upon reasonable request.
