# Supplementary material for: A Scalable and Robust Water Management Strategy for PEMFCs: Operando Electrothermal Mapping and Neutron Imaging Study
Source: Adv Sci (Weinh). 2024 Jul 25;11(36):2404350. doi: 10.1002/advs.202404350 (PMC11423223; doi:10.1002/advs.202404350)
Supplement: Supplementary file 1 — Supporting Information [file ADVS-11-2404350-s001.docx]

Supporting Information

A Scalable and Robust Water Management Strategy for PEMFCs: Operando Electrothermal Mapping and Neutron Imaging Study

Linlin Xu, Panagiotis Trogadas, Shangwei Zhou, Shuxian Jiang, Yunsong Wu, Lara Rasha, Winfried Kockelmann, Jia Di Yang, Toby Neville, Rhodri Jervis, Dan J. L. Brett, and Marc-Olivier Coppens*

Section S1. Simulation of capillary channel influence and calculation of optimal dimensions

The PEMFC computing domain in this study is shown in **Figures S1** and **S2**. The overall structure comprises cathode and anode flow channels with capillaries, gas diffusion layers (GDL), catalyst layers (CL), and membrane. The specific geometric and operating parameters are shown in **Table S1**.


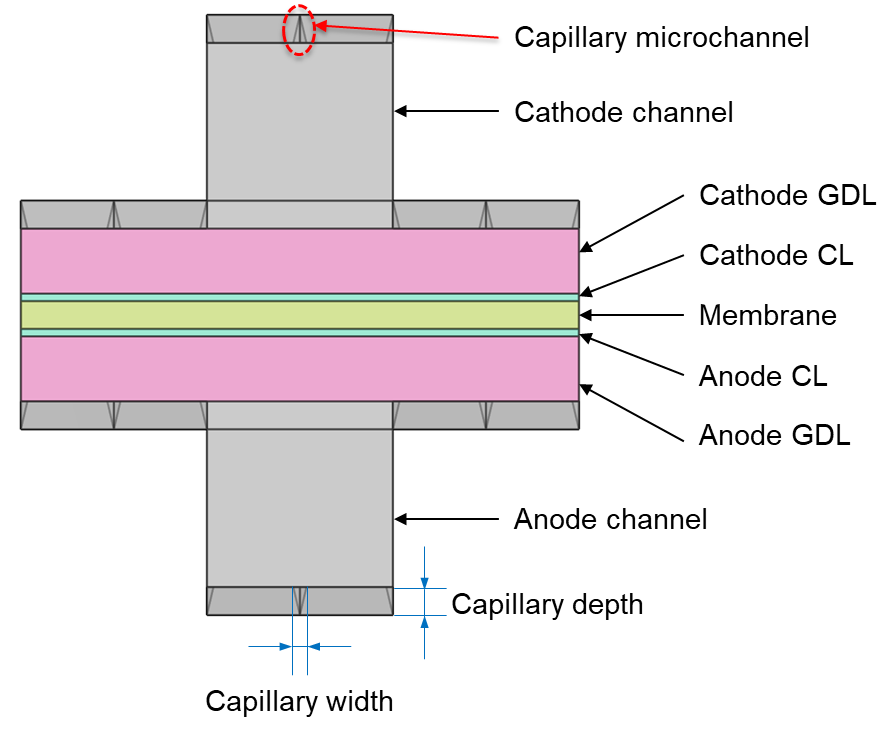


**Figure S1.** Geometrical schematic diagram of a PEMFC with lizard-inspired capillary microchannels.


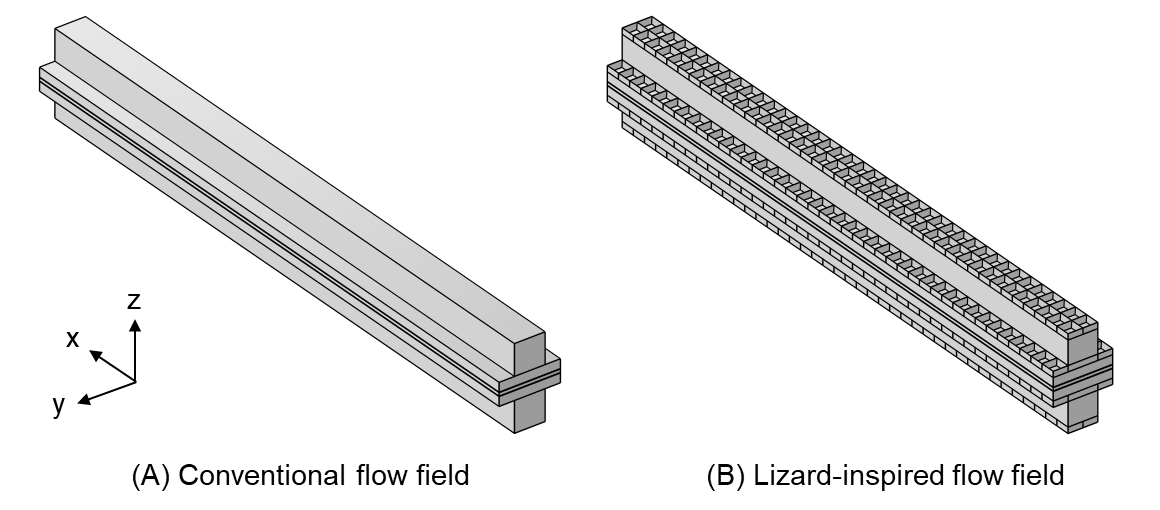


**Figure S2.** Computational domain of PEMFCs with (A) conventional flow field without capillaries and (B) lizard-inspired flow field with capillaries.

**Table S1.** Geometric and operating parameters.

| **Parameters** | **Value** | **Unit** | **Reference** |
| --- | --- | --- | --- |
| Cell length | 20 | mm |  |
| Channel height | 1 | mm | ^[1]^ |
| Channel width | 1 | mm | ^[1]^ |
| Rib width | 1 | mm | ^[1]^ |
| GDL thickness | 0.3 | mm | ^[1]^ |
| CL thickness | 0.0129 | mm | ^[1]^ |
| Membrane thickness | 0.108 | mm | ^[1]^ |
| GDL gas pore volume fraction (porosity) | 0.5 |  | ^[2]^ |
| Catalyst layer electrolyte (ionomer) volume fraction | 0.3 |  | ^[2]^ |
| Catalyst layer gas pore volume fraction | 0.5 |  | ^[2]^ |
| GDL permeability | 1.18×10^-11^ | m^2^ | ^[3]^ |
| Catalyst layer permeability | 2.36×10^-12^ | m^2^ | ^[3]^ |
| GDL electric conductivity | 222 | S/m | ^[4]^ |
| Membrane conductivity | 9 | S/m | ^[4]^ |
| Cell temperature | 353.15 | K | ^[4]^ |
| Reference pressure | 1 | atm | ^[1]^ |
| Cell voltage | 0.9 | V | ^[4]^ |
| Reference exchange current density, cathode | 1×10^-3^ | A/m^2^ | ^[5]^ |
| Reference exchange current density, anode | 1×10^2^ | A/m^2^ | ^[5]^ |
| Transfer coefficient, cathode | 1 |  | ^[5]^ |
| Transfer coefficient, anode | 0.5 |  | ^[6]^ |
| Specific surface area | 1×10^7^ | 1/m | ^[5]^ |
| Anode stoichiometry | 1.5 |  | ^[6]^ |
| Cathode stoichiometry | 3 |  | ^[6]^ |
| Relative humidity | 70 | % |  |
| Capillary width | 100 | µm |  |
| Capillary depth | 200 | µm |  |

To simplify the numerical simulation process, certain assumptions were made:^[7,8]^

- The fuel cell is operating at a steady state and gravity is ignored.
- Reactant gases behave as an ideal gas mixture.
- The flow in the gas channel is laminar.
- Water exists only in the vapor phase in the fuel cell.
- The diffusion layers and catalyst layers are isotropic and homogeneous.
- The membrane is fully humidified and impermeable to gas species.
- Three species, namely, oxygen, water, and nitrogen, are considered at the cathode side, while only hydrogen and water are considered at the anode side.

The domains were modelled using the finite element solver COMSOL Multiphysics 6.1, using Hydrogen Fuel Cell and Free and Porous Media Flow Multiphysics. Simulations were performed on a DELL Precision 5820 with 2x16 GB of RAM and a 3.2 GHz Intel Xeon processor. The governing equations in **Table S2** were used to define the fluid momentum in the porous medium, the movement of species from the flow field to the catalyst layer, and the reactions and currents generated in the catalyst layer.

**Table S2.** Governing equations of the numerical model of PEMFC.^[3,8,9]^

| **Governing Equation** | **Source Term Specification** |
| --- | --- |
| Butler-Volmer equation (CLs):  $i_{loc}=i_{0}\left[ exp\left( \frac{\alpha_{a}F\eta}{RT} \right)-exp\left( \frac{\alpha_{c}F\eta}{RT} \right) \right]$ | $\eta=E_{ct}-E_{eq}$  $E_{ct}=\phi_{s}-\phi_{l}$ |
| Ohm’s law (Membrane, GDLs and CLs):  $i_{k}={-\sigma}_{k}\nabla\phi_{k}$ | $\nabla i_{k}=Q_{k}$ |
| Maxwell-Stefan equation (Flow channels, GDLs and CLs):  $\rho\left( u\cdot\nabla\right)\omega_{i}={\nabla\cdot\left( \rho\omega_{i}\sum_{k} \tilde{D}_{ik}d_{k} \right)+R}_{i}$ | $d_{k}=\nabla x_{k}+\frac{1}{p}\left[ \left( x_{k}-\omega_{k} \right)\nabla p \right]$  $x_{k}=\frac{\omega_{k}}{M_{k}}M_{n}$  $M_{n}=\left( \sum_{i} \frac{\omega_{i}}{M_{i}} \right)^{-1}$ |
| Navier-Stokes equation (Flow channels):  $\rho\left( u\cdot\nabla\right)u=\nabla\cdot\left( -pI+K \right)+F$ | $\nabla\cdot\left( \rho u \right)=0$  $K=\mu\left[ \nabla u+\left( \nabla u \right)^{T} \right]-\frac{2}{3}\mu\left( \nabla\cdot u \right)I$ |
| Brinkman equation (GDLs and CLs):  $\frac{1}{\varepsilon_{p}}\rho\left( u\cdot\nabla\right)u\frac{1}{\varepsilon_{p}}=\nabla\cdot\left[ -pI+K \right]-\left( \mu\kappa^{-1}+\beta\rho\left\vert u \right\vert+\frac{Q_{m}}{\varepsilon_{p}^{2}} \right)u+F$ | $\nabla\cdot\left( \rho u \right)=Q_{m}$  $K=\mu\frac{1}{\varepsilon_{p}}\left[ \nabla u+\left( \nabla u \right)^{T} \right]-\frac{2}{3}\mu\frac{1}{\varepsilon_{p}}\left( \nabla\cdot u \right)I$ |

Before the numerical simulations, the following boundary conditions were set for the models:^[1,4,10]^

- Symmetric boundary conditions for the gas diffusion layers and catalyst layers.
- No slip condition.
- All initial values are set to zero.
- Reference pressure is set to 1 atm.
- The flow channel outlet is set as pressure boundary with atmospheric pressure.
- The inlet of the flow channel is set to the velocity boundary. Inlet velocities for the anode and cathode can be calculated as follows:

$$U_{in,a}=\zeta_{a}\frac{I_{stoi,a}}{2F}\frac{1}{x_{H_{2}}}\frac{RT}{p_{ref}}\frac{1}{A_{ch}}$$

$$U_{in,c}=\zeta_{c}\frac{I_{stoi,c}}{4F}\frac{1}{x_{O_{2}}}\frac{RT}{p_{ref}}\frac{1}{A_{ch}}$$

where $U_{in}$ is the average inlet velocity, $\zeta$ is the stoichiometric ratio, $I_{stoi}$ is the operating current for gas flow calculations, $x_{H_{2}}$ is the hydrogen mole fraction, $x_{O_{2}}$ is the oxygen mole fraction, $A_{ch}$ is the total cross-sectional area of flow channels, and the subscripts a and c represent the anode and cathode, respectively.

First, an analysis of the effect of mesh size for the PEMFC model with conventional flow field was carried out to reduce the calculation cost and ensure mesh-independent simulation results. The mesh number and current density at 0.4 V are shown in **Figure S3**. Also, the relative difference in current density for different meshing schemes was calculated. The current density result has less than 0.15% error when the number of mesh elements is above 92,340 (star), indicating that the solution is mesh-independent at this level of fineness.


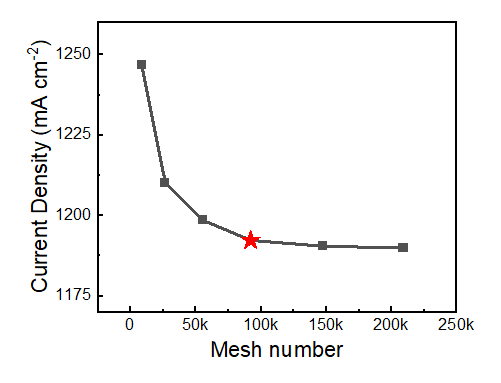


**Figure S3.** Grid independence test.

In this study, model validation was performed by comparing the polarisation curve obtained from the numerical simulations and the experimental results on a 25 cm^2^ parallel flow field based PEMFC under the same conditions. From **Figure S4**, it can be observed that the simulation results agree with the experimental data. Consequently, the proposed model has been experimentally validated.


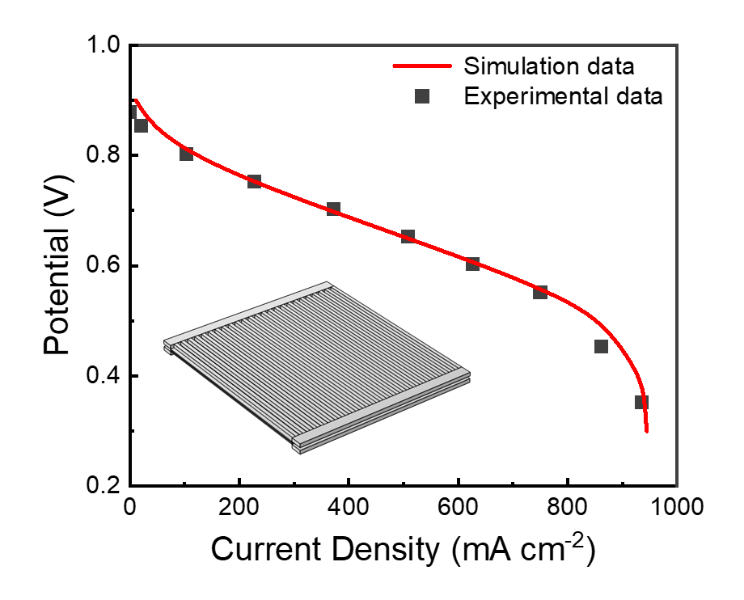


**Figure S4.** Model validation: comparison of polarisation curve between simulations and experiments. PEMFC scale: 25 cm^2^, cell temperature: 80 °C, RH: 70%, the stoichiometric ratio of hydrogen and air: 1.5 and 3, respectively.

PEMFC performance and operation stability are significantly affected by the oxygen distribution at the interface between GDL and CL on the cathode side.^[11]^ **Figure S5** shows the mole fraction of oxygen in the flow channel and at the interface between cathode GDL and CL at 0.4 V cell potential. It is shown that the oxygen mole fraction is continuously decreasing from the inlet to the outlet because of the consumption of reactants in the electrochemical reactions, which is negatively correlated with the water distribution (**Figure S6**). Both flow fields share the same oxygen mole fraction at the inlet of the flow channel. As O_2_ flows in the flow channel and diffuses through the GDL, the lizard-inspired flow field can provide more oxygen (Figure S5). This indicates that the lizard-inspired flow field, with increased surface area and volume available for gas phase flow, has superior mass transfer to the conventional channel. This is reflected not only in the transfer and distribution of O_2_ but also in water removal. As shown in Figure S6, water generated from the lizard-inspired flow field in the GDL is much lower than in the counterpart. In the case of the conventional flow field, a deficiency in local oxygen concentration and water blockage occurred beneath the land area. Persistent local water blockage, if not addressed, is more prone to diminishing the performance and durability of the fuel cell due to flooding.^[8]^ The membrane current density distribution in different flow fields at 0.4 V is illustrated in **Figure S7**. It can be observed that the higher and more uniform distribution of current density appears in the lizard-inspired flow field since the capillary network enhances the reactant mass transfer (Figure S5) and water removal (Figure S6). These simulation results strongly indicate that incorporating capillaries in flow fields plays a crucial role in enhancing the performance of PEMFCs.


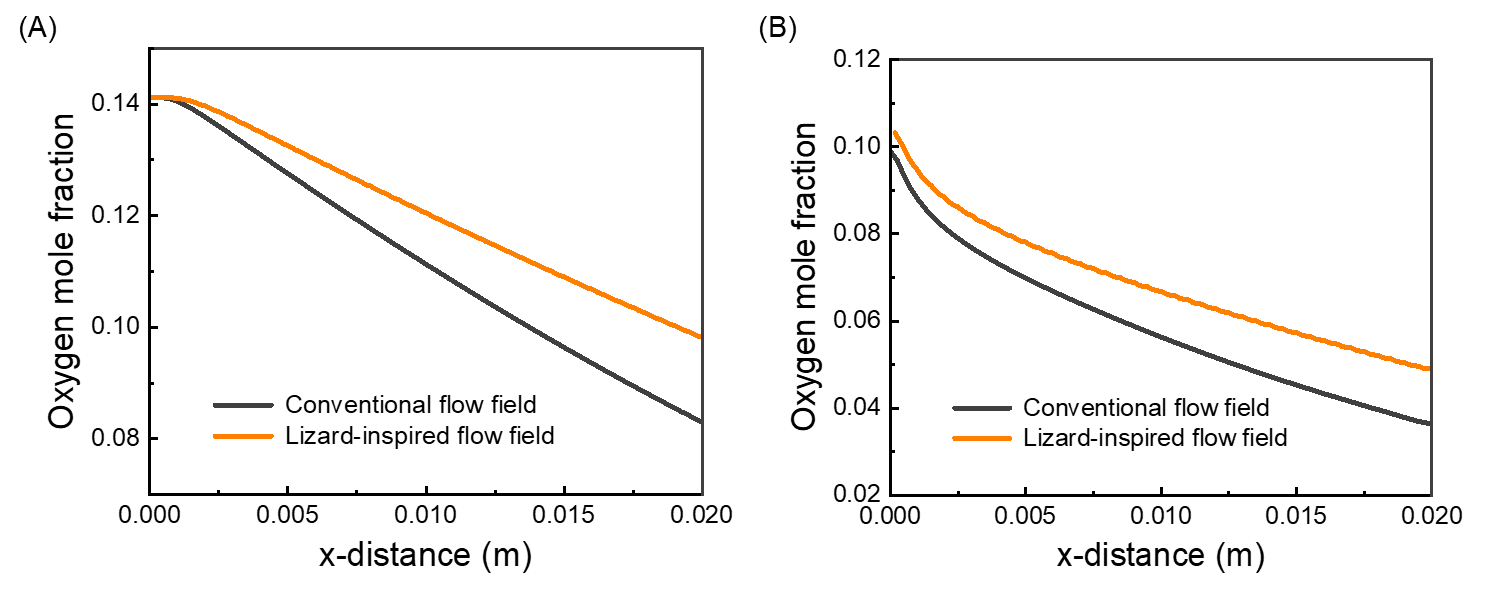


**Figure S5.** Oxygen mole fraction (A) along the central line of the cathode channel and (B) along the middle line of the interface between cathode GDL and CL at 70% RH, 80 °C, and 0.4 V.


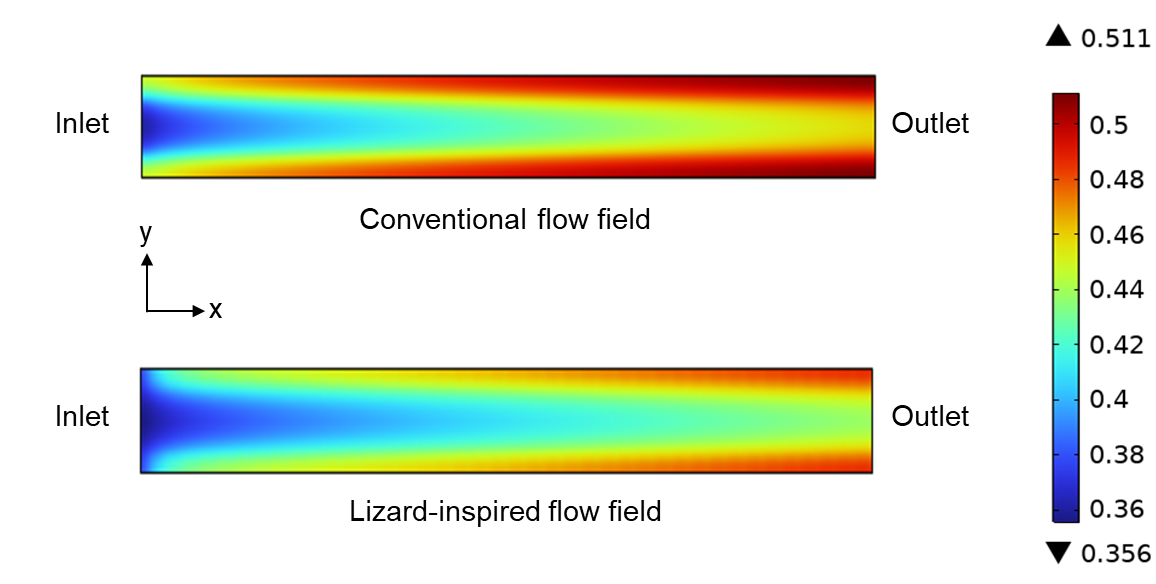


**Figure S6.** Water mole fraction distribution in cathode GDL at 70% RH, 80 °C, and 0.4 V.


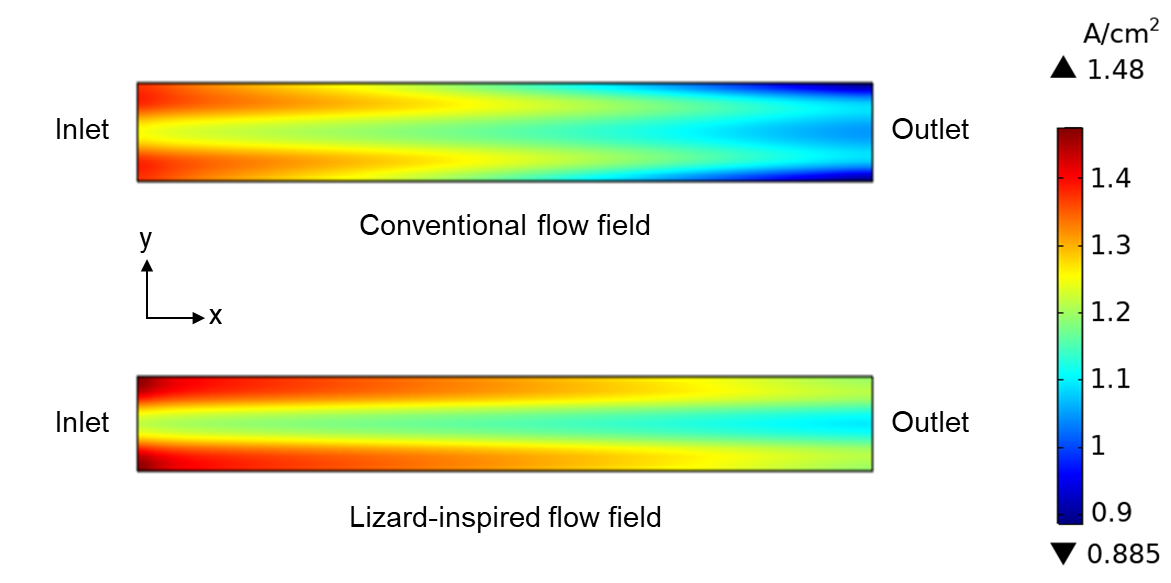


**Figure S7.** Current density distribution in the membrane at 70% RH, 80 °C, and 0.4 V.

In-depth investigations into the impact of capillary channel width and depth on PEMFCs can reveal significant insights for practical processing of the lizard-inspired flow field. The interplay between channel dimensions and performance is intricate, with narrower and deeper capillary channels significantly enhancing PEMFC performance (**Figure S8**). However, as the width diminishes, the rise in current density decelerates, particularly when the width falls below 100 µm, with the increment in current density remaining within a 0.5% range. Therefore, in the practical manufacturing of lizard-inspired flow fields, it is advisable to maximize the capillary depth, ensuring that the capillary width remains within the range of 100 µm.


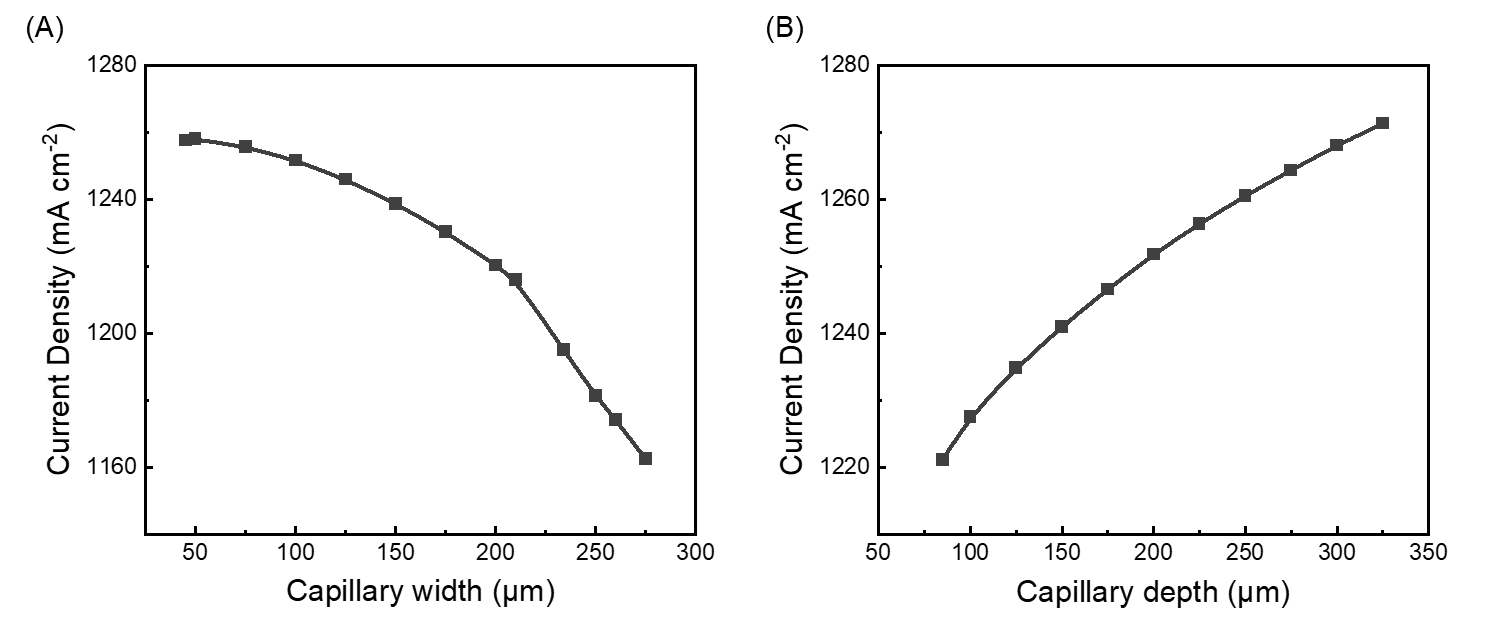


**Figure S8.** PEMFC performance comparison of flow fields with capillaries. Effect of (A) capillary width and (B) capillary depth on current density at 70% RH, 80 °C, and 0.4 V.

**Section S2. Flow field design**


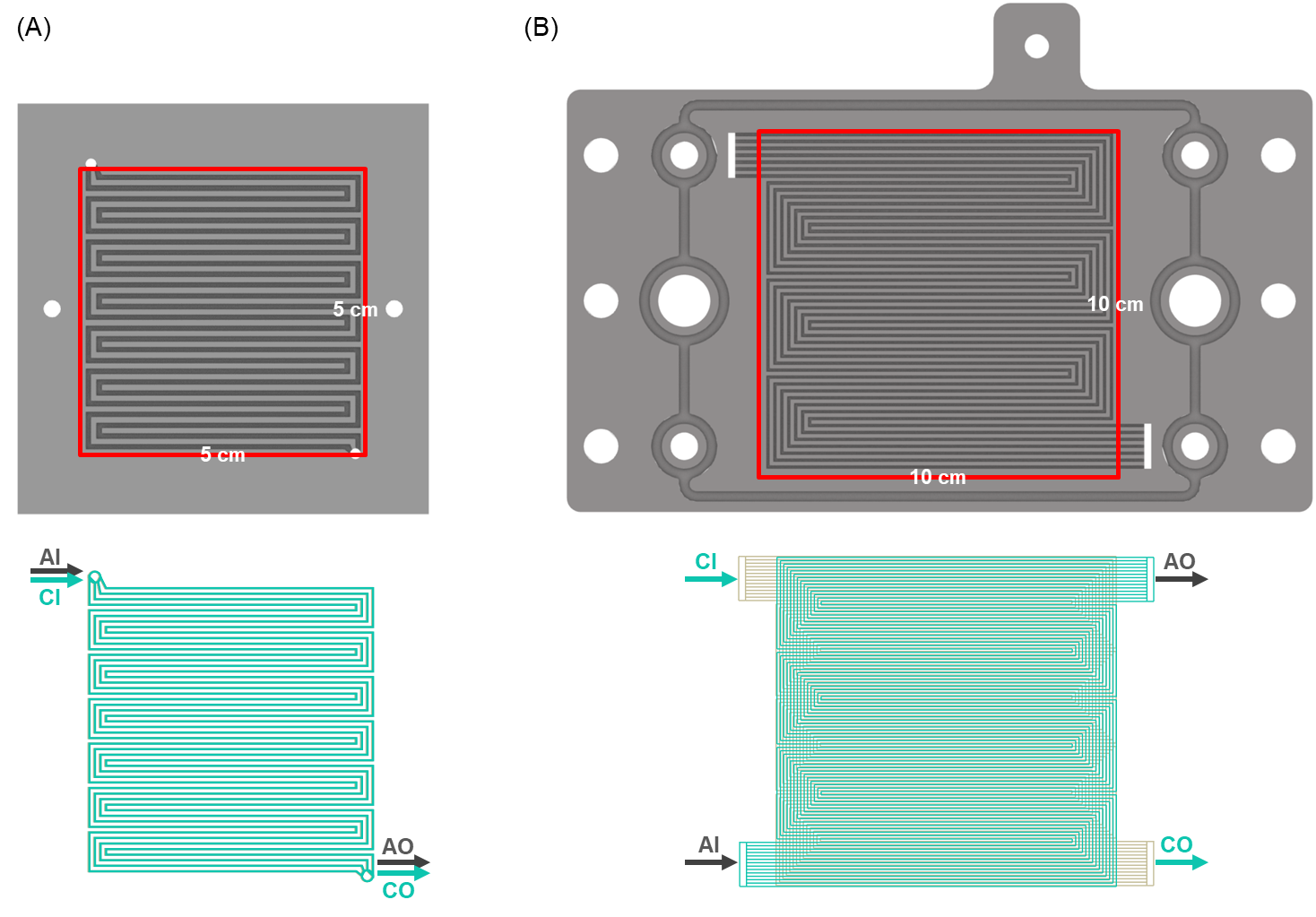


**Figure S9.** Flow field designs and flow configurations of (A) 25 cm^2^ double-serpentine flow field (co-current) and (B) 100 cm^2^ septuple-serpentine flow field (counter-current). The red box represents the active area. The double-serpentine flow field consists of two parallel serpentine (snake-like) channels and the septuple-serpentine flow field features seven parallel serpentine channels.

**Section S3. Durability test**

**Figure S10.** The potential variation of lizard-inspired double-serpentine flow field at 100% RH under 1000 mA cm^−2^.

**Section S4. Measurement of the attenuation coefficient of water**

A water container (**Figure S11**) with a known thickness of 1 mm was placed 100 mm from the neutron scintillator screen to test the attenuation coefficient of water at different flux levels, which was achieved by using three ‘pinholes’ (PH), i.e. circular aperture diameters of 20 mm (PH20), 30 mm (PH30), and 40 mm (PH40), respectively (**Figure S12**). The attenuation coefficient of water, *µ*, was calculated from the relative neutron transmission (*I / I_0_*) by inverting the Beer-Lambert law (**Table S3**):^[12]^

$$\mu=-\frac{\ln\left( I/{I_{o}} \right)}{t} [S1]$$

where *I* is the intensity of the beam in operation, *I_0_* is the intensity of the beam of the dry PEMFC, and *t* is the thickness corresponding to water presence.


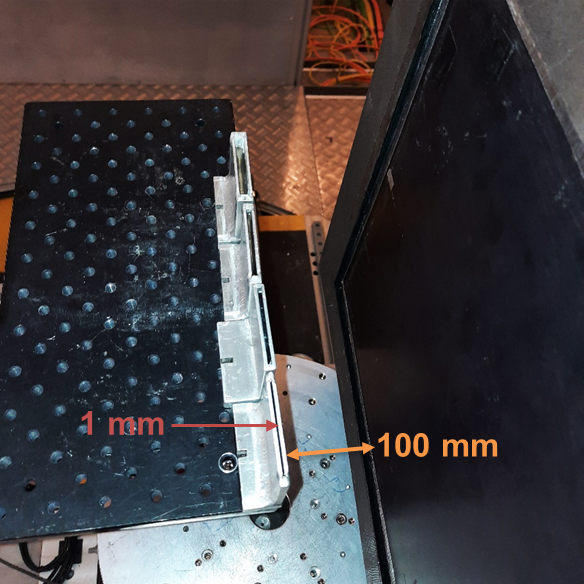


**Figure S11.** Experimental setup with a 1 mm thick water container to measure the attenuation coefficient.


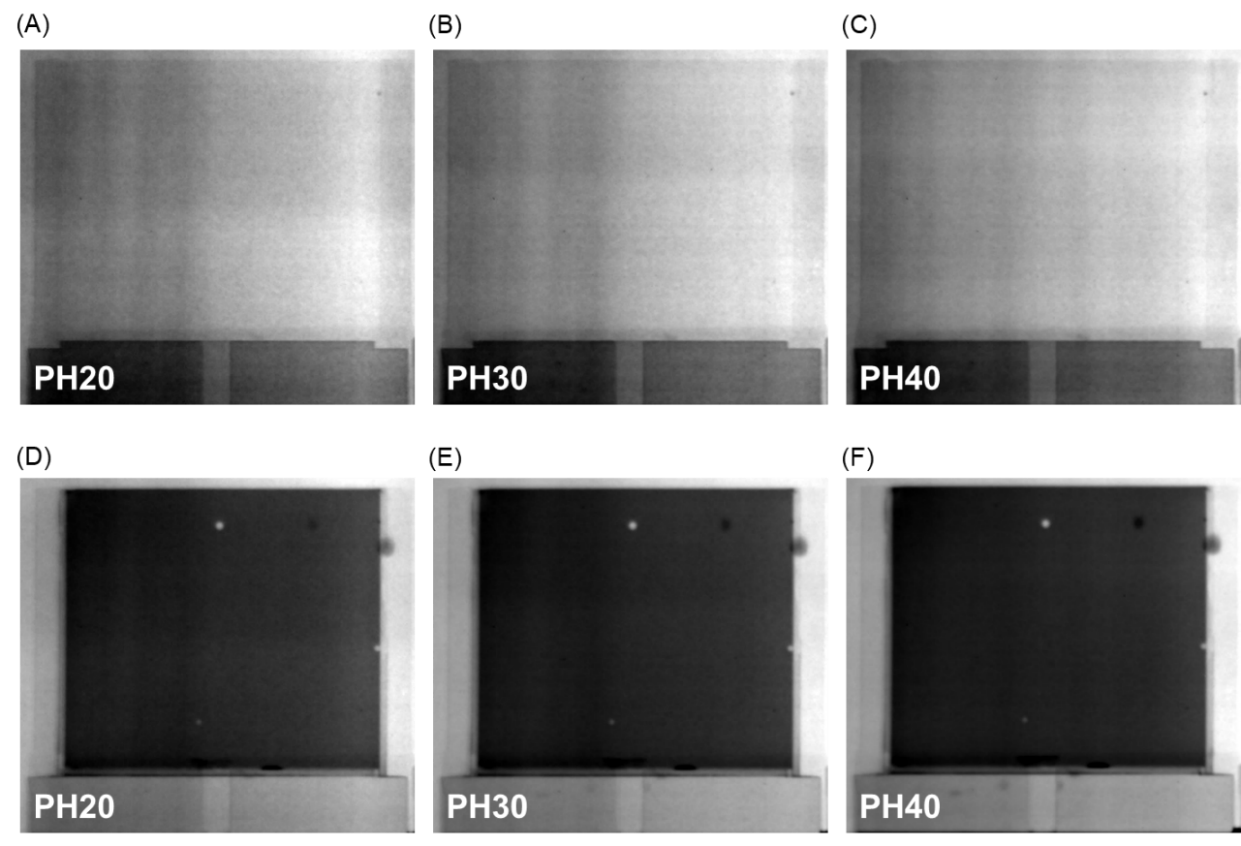


**Figure S12.** Radiograph of a (A-C) dry container and (D-F) 1 mm thick water-containing container at (A, D) PH20, (B, E) PH30, and (C, F) PH40.

**Table S3.** The attenuation coefficient of water calculated at PH20, PH30, and PH40.

|  | ***t*** / cm | **-ln (*I / I_0_*)** ^a^ | ***µ*** / cm^-1^ |
| --- | --- | --- | --- |
| **PH20** | 0.1 | 0.53 | 5.3 |
| **PH30** | 0.1 | 0.53 | 5.3 |
| **PH40** | 0.1 | 0.53 | 5.3 |

^a^ -ln (*I / I_0_*) is calculated using MATLAB.

Section S5. Water distribution in a 25 cm^2^ fuel cell


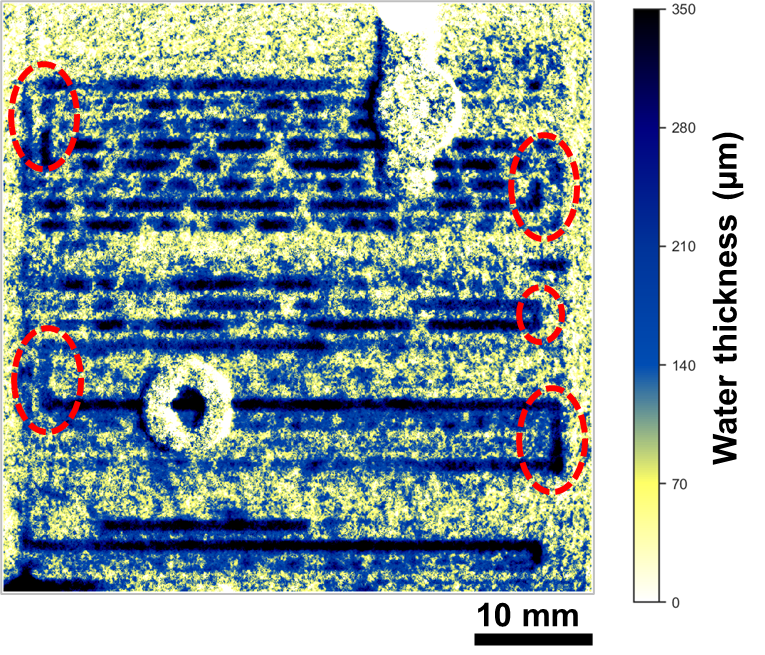


**Figure S13.** Water thickness distribution in a 25 cm^2^ double-serpentine flow field-based PEMFC after 750 s of galvanostatic operation at 400 mA cm^-2^. Experiments were conducted at a cell temperature of 40 ºC using dry hydrogen and dry air at a stoichiometric ratio of 2 and 3, respectively.

Section S6. Pressure drop


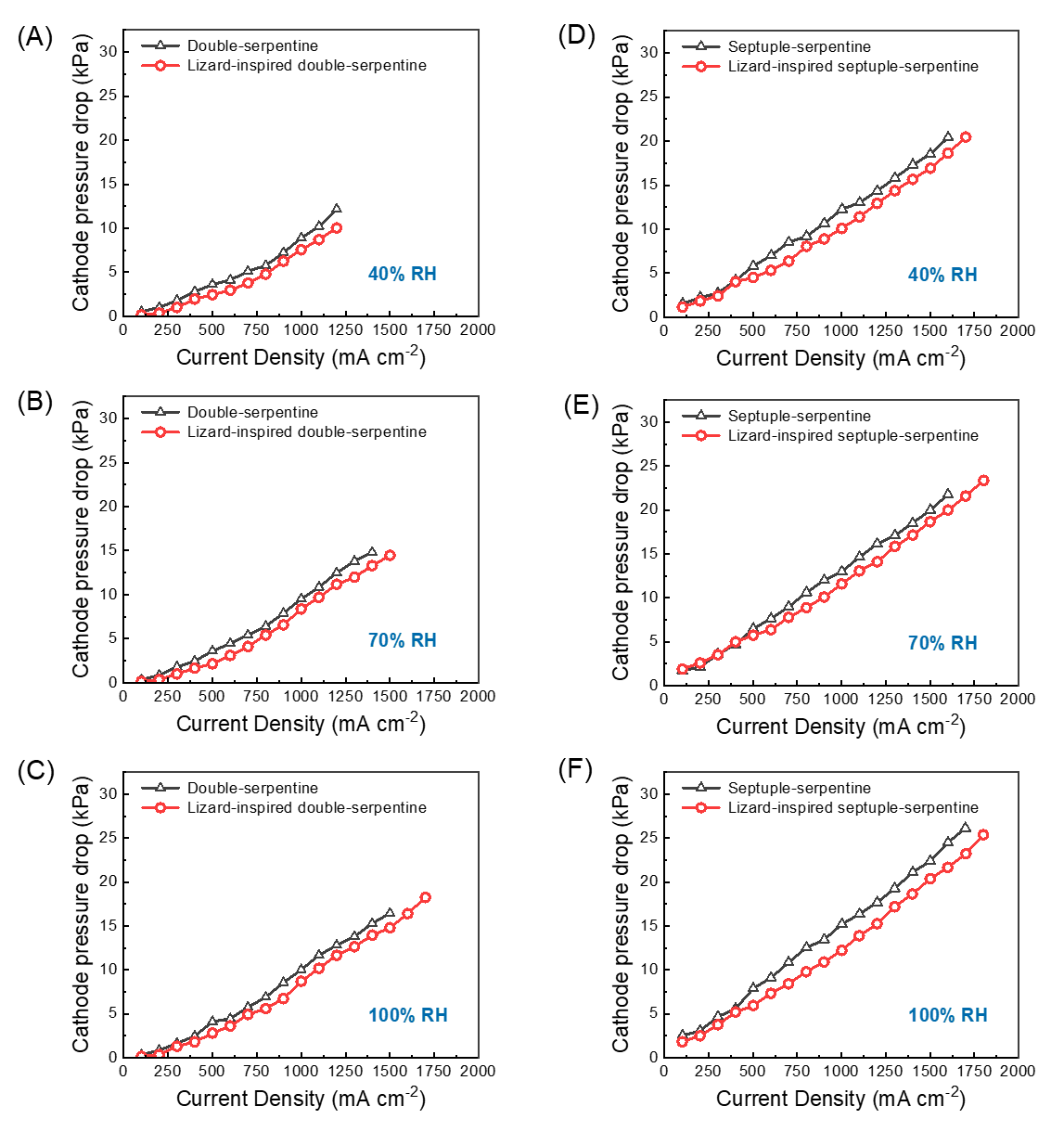


**Figure S14.** Comparison of pressure drop in the cathode for (A-C) 25 cm^2^ cells and (D-F) 100 cm^2^ cells at (A, D) 40% RH, (B, E) 70% RH, and (C, F) 100% RH, respectively.

**References**

[1] F. Sun, D. Su, Y. Yin, *Int. J. Energy Res.* **2023**, *2023*, 1.

[2] Y. Yang, M. Bai, Z. Zhou, W.-T. Wu, C. Hu, L. Gao, Y. Li, Y. Li, Y. Song, *Int. J. Heat Mass Transfer* **2024**, *218*, 124793.

[3] S. Mohanty, A. N. Desai, S. Singh, V. Ramadesigan, S. M, *Int. J. Hydrogen Energy* **2021**, *46*, 20650.

[4] F. Yan, X. Pei, J. Yao, *Ionics* **2023**, *29*, 695.

[5] Y. Sun, L. Mao, H. Wang, Z. Liu, S. Lu, *Int. J. Hydrogen Energy* **2022**, *47*, 33439.

[6] X. Deng, E. Zhang, J. Lei, D. Jia, Y. Liu, H. E. Shuchao, *ACS Omega* **2022**, *7*, 42872.

[7] Z. Zhang, Q. Wang, F. Bai, L. Chen, W. Tao, *Energy* **2023**, *263*, 125897.

[8] D. Park, S. Ham, Y.-J. Sohn, Y.-Y. Choi, M. Kim, *Int. J. Hydrogen Energy* **2023**, *48*, 304.

[9] O.-J. Kwon, H.-S. Shin, S.-H. Cheon, B. S. Oh, *Int. J. Hydrogen Energy* **2015**, *40*, 11577.

[10] J. Song, H. Guo, F. Ye, C. F. Ma, *Int. J. Energy Res.* **2019**, *43*, 2940.

[11] S. Guo, Y. Zhao, C. Pan, X. Wang, T. Xu, *Int. J. Hydrogen Energy* **2023**, *48*, 36937.

[12] J. I. S. Cho, T. P. Neville, P. Trogadas, Q. Meyer, Y. Wu, R. Ziesche, P. Boillat, M. Cochet, V. Manzi-Orezzoli, P. Shearing, D. J. L. Brett, M. O. Coppens, *Energy* **2019**, *170*, 14.

**Nomenclature**

| CL | catalyst layer |
| --- | --- |
| D | diffusivity, m^2^ s^-1^ |
| E | cell potential, V |
| F | Faraday constant, C mol^-1^ |
| GDL | gas diffusion layer |
| i | current density, A m^-2^ |
| i_0_ | exchange current density, A m^-2^ |
| k | permeability, m^2^ |
| M | molar mass, kg mol^-1^ |
| p | pressure, Pa |
| Q_m_ | mass source |
| R | universal gas constant, J mol^-1^ K^-1^ |
| R_i_ | source term due to the reaction, kg m^-3^ s^-1^ |
| RH | relative humidity, % |
| T | temperature, K |
| u | velocity, m s^-1^ |
| w | mass fraction |
| x | mole fraction |

**Greek**

| α | charge transfer coefficient |
| --- | --- |
| β | Forchheimer drag coefficient |
| ε | porosity |
| φ | potential, V |
| η | overpotential, V |
| ρ | density, kg m^-3^ |
| σ | effective electric conductivity, S m^-1^ |
| τ | tortuosity |

**Superscripts and subscripts**

| 0 | intrinsic |
| --- | --- |
| a | anode |
| c | cathode |
| eq | equilibrium |
| i | species i |
| in | inlet |
| k | species k |
| l | electrolyte |
| loc | local |
| out | outlet |
| s | electrode |
